# First description of the male of *Solenura ania* (Walker) (Hymenoptera: Pteromalidae), a giant pteromalid parasitoid of *Trichoferus campestris* (Faldermann), with special reference to its sexual dimorphism

**DOI:** 10.3897/BDJ.8.e54961

**Published:** 2020-07-29

**Authors:** Liangming Cao, Jianxin Cui, Xiaoyi Wang, Guisen Wang, Zhongqi Yang

**Affiliations:** 1 Key Laboratory of Forest Protection of National Forestry and Grassland Administration, Research Institute of Forest Ecology, Environment and Protection, Chinese Academy of Forestry, Beijing, China Key Laboratory of Forest Protection of National Forestry and Grassland Administration, Research Institute of Forest Ecology, Environment and Protection, Chinese Academy of Forestry Beijing China; 2 Department of Plant Protection, Henan Institute of Science and Technology, Xinxiang, China Department of Plant Protection, Henan Institute of Science and Technology Xinxiang China; 3 Shunwang Street Agricultural Comprehensive Service Center, Zhucheng City, China Shunwang Street Agricultural Comprehensive Service Center Zhucheng City China

**Keywords:** Palaearctic, ectoparasitoid, China, Lyciscini

## Abstract

**Background:**

The giant pteromalid wasp *Solenura ania* (Walker) has a distinct sexual dimorphism. The metallic blue female is about 28 mm long and the metallic green male is only about 6 mm in length. This species is an ectoparasitoid of some woodborers, such as *Trichoferus campestris* (Faldermann), a pest of many live trees and wood furniture and a quarantine pest in many countries. However, the male of this pteromalid was not described prior to this study.

**New information:**

The male of the species is first described, based on newly-collected material. Photographs of habitus, head, mesosoma, metasoma and other structures of both sexes are provided to facilitate recognition of this giant pteromalid. Sexual dimorphism is also compared in the present study.

## Introduction

Pteromalidae is one of largest families in Hymenoptera, including 588 genera and more than 3500 species placed in 31 subfamilies (Noyes 2019). *Solenura* Westwood, 1868 includes brightly coloured species with large body size, whose female individuals can reach nearly 30 mm in length, 5 times as long as male individuals. Specimens of *Solenura*, especially males, are relatively rare in museum collections. Therefore, sexual dimorphism in this genus is seldom mentioned, which is representative and extreme in this family or even in Chalcidoidea. In this study, we describe in detail and illustrate with colour microphotographs the male and sexual dimorphism in *Solenura ania* (Walker).

## Materials and methods

This study is based on specimens preserved in the Entomological Museum of Chinese Academy of Forestry. Specimens were examined with an SZH 1500 stereomicroscope (Nikon, Tokyo, Japan). Photographs of the specimens were taken with a CX31 microscope (Olympus, Tokyo, Japan) with the UV–C Optical Totally Focuses System developed by Beijing United Vision Technology Co. Ltd. (Beijing, China). Terminology follows Gibson (2003). Measurements were obtained using a calibrated micrometer.

## Taxon treatments

### Solenura ania

(Walker, 1846)

765CF520-6C57-5968-A2EE-064FDAE73576

#### Materials

- **Type status:** Other material. **Occurrence:** individualCount: 1; sex: male; lifeStage: adult; **Taxon:** scientificName: Solenura ania; acceptedNameUsage: Solenura ania; parentNameUsage: Solenura ania; order: Hymenoptera; family: Pteromalidae; genus: Solenura; **Location:** country: China; stateProvince: Henan; locality: Tianjie Mountain; verbatimLatitude: 35°35′11.53″N; verbatimLongitude: 113°36′5.08″E; **Identification:** identifiedBy: Liangming Cao; dateIdentified: 2019; **Event:** eventDate: 2018-07-1; **Record Level:** language: English; collectionCode: Insects; basisOfRecord: PreservedSpecimen
- **Type status:** Other material. **Occurrence:** individualCount: 1; sex: female; lifeStage: adult; **Taxon:** scientificName: Solenura ania; acceptedNameUsage: Solenura ania; parentNameUsage: Solenura ania; order: Hymenoptera; family: Pteromalidae; genus: Solenura; **Location:** country: China; stateProvince: Henan; locality: Tianjie Mountain; verbatimLatitude: 35°35′11.53″N; verbatimLongitude: 113°36′5.08″E; **Identification:** identifiedBy: Liangming Cao; dateIdentified: 2019; **Event:** eventDate: 2018-07-1; **Record Level:** language: English; collectionCode: Insects; basisOfRecord: PreservedSpecimen

#### Description

**Description of male.** Body length 5.92 mm, length of fore wing 3.23 mm (Fig. 2a).

**Figure 1.**
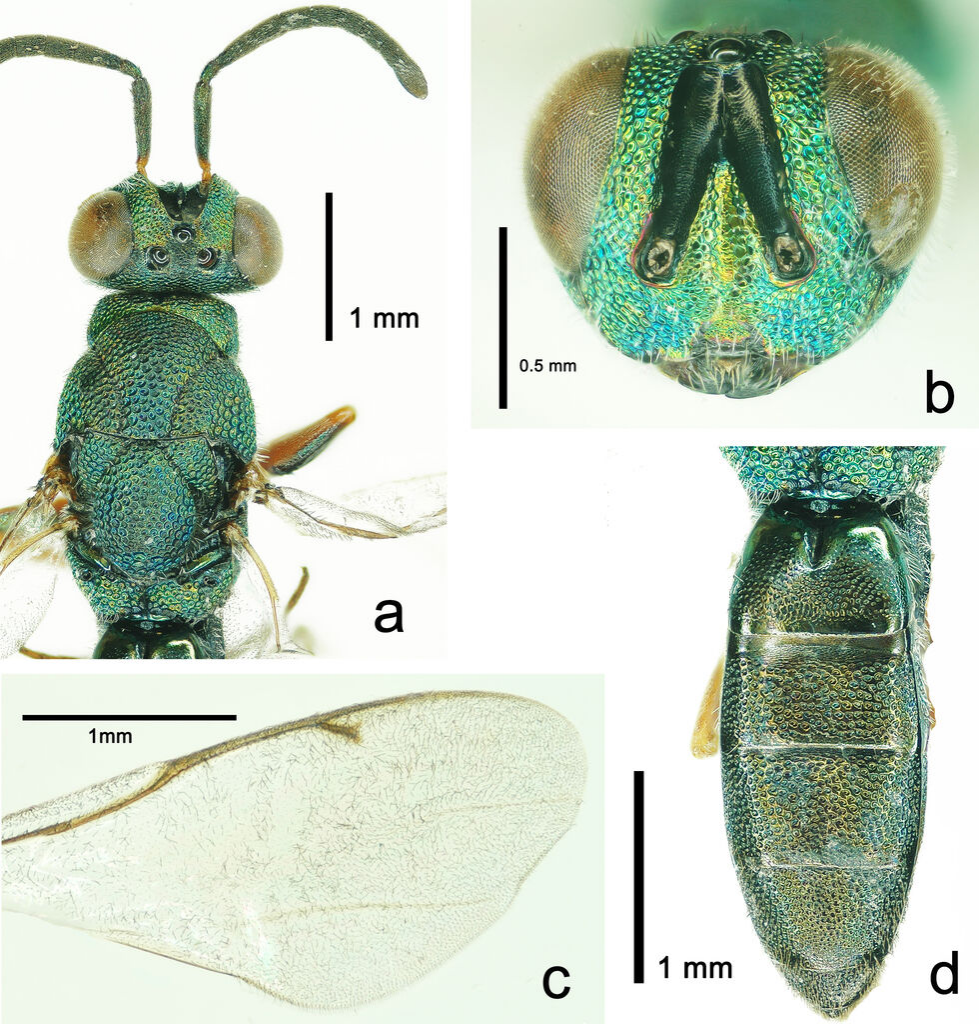
*Solenura ania* (Walker), male. **a.** Head and mesosoma in dorsal view; **b.** head in frontal view; **c.** fore wing; **d.** metasoma in dorsal view.

**Figure 2.**
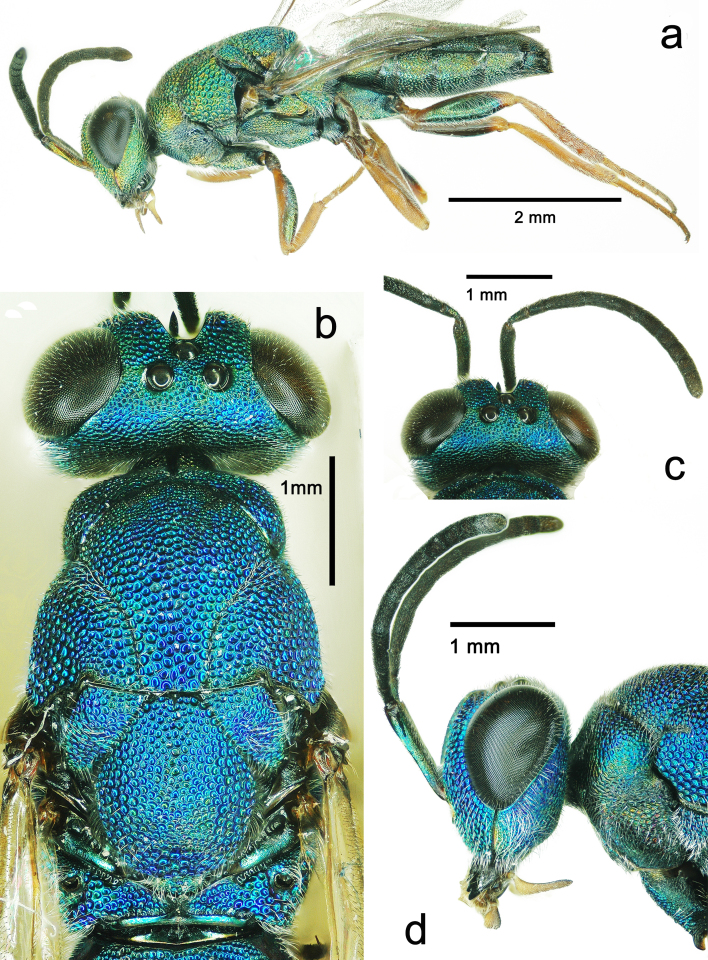
*Solenura ania* (Walker). **a.** male, lateral habitus; **b.** female, head and mesosoma in dorsal view; **c.** female, antenna in dorsal view; **d.** female, head in lateral view.

**Colour**: Body green, with metallic tint (Fig. 2a). Head with ocelli, scrobal depression and apical 2/3 mandibles black, interantennal triangular region golden, eyes brown (Fig. 1b); antennal radicle and basal apex of scape orange; labial and maxillary palpi, dorsal side of fore, mid and hind femora and entire tibiae and tarsi orange to reddish-brown, ventral side of all femora metallic green (Fig. 2a); both wings subhyaline, veins and setae dark brown.

**Head.** In dorsal view, head 1.86 times as broad as long; eyes large, width 0.3 times maximum width of head, interantennal triangular region sharp, ocelli large, anterior ocellus with frontal margin touching scrobal depression, ocello-ocular distance 0.5 times post-ocellar distance (Fig. 1a). In frontal view, head 1.2 times as broad as high, eyes 0.65 times head height, frons in narrowest part twice eye breadth; frons and face with strong reticulation; scrobes deep, groove-like, reaching the anterior ocellus and in the form of an inverted V, with scale-like sculpture; outer margin of scrobes parallel with inner margin of eyes; malar sulcus deep, slightly curved; mandibles with two blunt teeth; antennal toruli same level with lower eye margin (Fig. 1b). Malar space 0.55 times eye height, posterior margin of eye touching occiput, thus temple undeveloped (Fig. 1a, Fig. 2a). Antennal radicle 0.13 times scape length; scape slightly curved and broadening in apical half, 3.75 times as long as pedicel, 1^st^ funicular segment 0.5 times 2^nd^ funicular segment; clava 1.6 times as long as last funicular segment.

**Mesosoma.** Mesosoma with conspicuous and strong punctate reticulation. Pronotal collar quadrate, anterior margin straight, with dense reticulation. Pronotal collar 0.08 times as long as mesoscutum and 0.75 times as long as broad. Mesoscutum 0.65 times as long as broad, mid lobe convex, notauli deep. Axillae large. Scutellum conspicuously convex, 1.3 times as broad as long. Propodeum short, medially 0.25 times length of scutellum, median carina obvious and straight, median area shiny with strong punctuate reticulation; spiracles oval (Fig. 1a). In lateral view, prepectus delicately reticulate, with long hairs; mesopleuron setose, reticulate to punctate-alveolate; metapleuron shiny and densely haired (Fig. 2a). Fore leg with femur slightly curved and 1.6 times as long as tibia, tarsus 1.38 times as long as tibia, tibia with one spur, which is 0.6 times as long as basitarsus, tarsal segments 1-5 with relative length of 40:30:25:30:28; mid leg with femur slightly bulgy, 0.95 times as long as tibia, tarsus as long as tibia, tarsal segments 1-5 with relative length of 15:15:10:8:8; hind leg with femur 4.1 times as long as broad, tibia 1.05 times as long as femur, ventrally with two equally long apical spurs, the spurs 0.5 times as long as basitarsus, tarsus 0.77 times as long as tibia, tarsal segments 1-5 with relative length of 25:25:15:12:15.

**Wing.** Fore wing nearly reaching the apex of gaster, disc with dense setae; submarginal vein twice as long as marginal vein, marginal vein 1.8 times as long as postmarginal vein and 3.6 times as long as stigmal vein, R vein and Cu vein faint but visible (Fig. 1c). Hind wing about 0.7 times as long as fore wing.

**Metasoma.** Metasoma sessile with gaster 0.85 times as long as head plus mesosoma; gaster punctuate reticulation. Posterior margins of tergites 1, 3, 4 straight, of tergite 2 concave, of tergite 5, 6 convex; 1^st^ tergite with V-like basal cavity occupying 0.33 times tergite median length, 1.33 times as long as 2^nd^ tergite and 1.23 times as long as 3^rd^ tergite; 4^th^ tergite 1.2 times as long as 5^th^ tergite (Fig. 1d).

**Figure 3.**
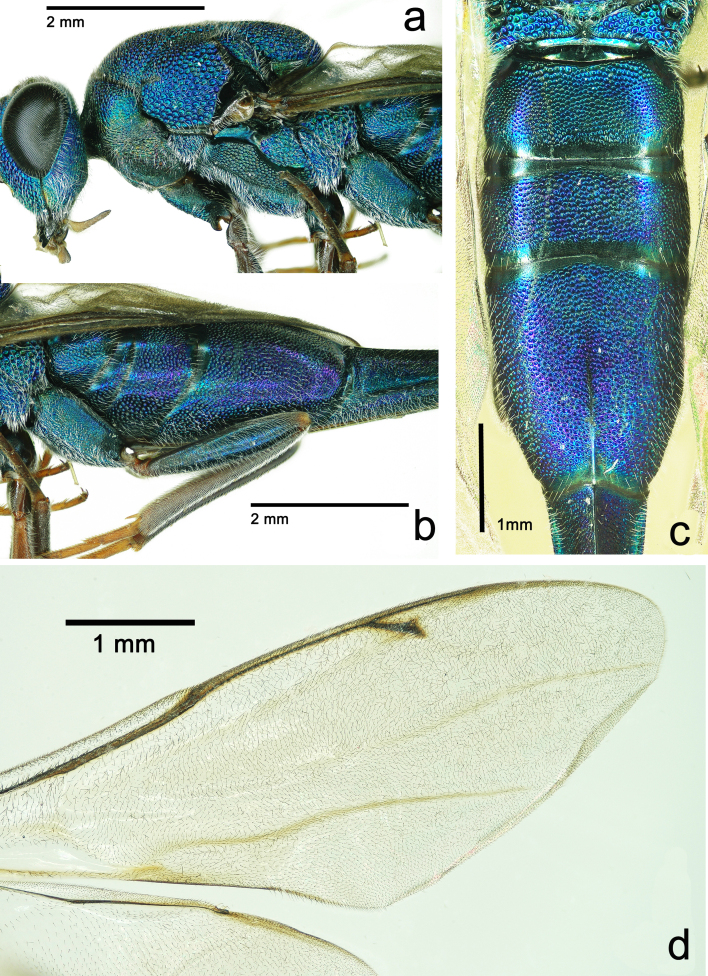
*Solenura ania* (Walker), female. **a.** Mesosoma in lateral view; **b.** basal 3 tergites of metasoma in lateral view; **c.** basal 3 tergites of metasoma in dorsal view; **d.** fore wing.

**Figure 4.**
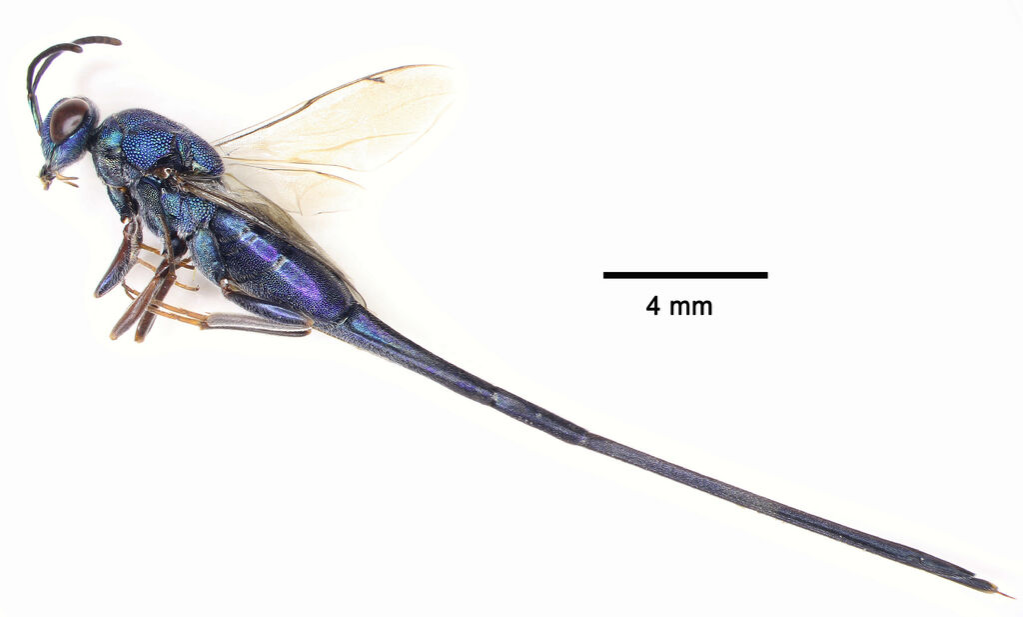
*Solenura ania* (Walker), female, habitus in lateral view.

**Female.** (Figs 2, 3, 4) The female has been re-described in several previous studies (Matsumura 1912, Cameron 1909, Girault 1927, Yang 1991, Huang and Xiao 2005, Sureshan 2005).

#### Distribution

China [Henan (new record), Shaanxi (Yang 1991), Anhui (Gibson 2003), Beijing (Huang and Xiao 2005), Jiangsu (Gibson 2003), Liaoning (Huang and Xiao 2005), Shandong (Gibson 2003), Taiwan (Matsumura 1912)]; India; Indonesia; Japan; Malaysia; Philippines; Sri Lanka; Singapore; Thailand (Noyes 2019).

#### Biology

**Hosts.** Buprestidae: *Chrysobothris succedanea* Saunders (Yang 1991); Cerambycidae: *Clytocera chinospila* Gahan, *Olenecamptus bilobus* (Fabricius) (Sureshan et al. 2013), *Trichoferus campestris* Faldermann (Yang 1991).

#### Taxon discussion

**History.** Walker (1846) described only the female of *Epistenia ania*, stating that the body colour is “purple” and providing no figures. Matsumura (1912) described the female of *Thecasoma longicauda* from Taiwan Province, with a habitus figure. Cameron (1909) described the female of *Taoga rufipes*, stating a body length of only 14 mm. Girault (1927) described only the female of *Thaumasurelloides silvae*. Boućek et al. (1979) designated lectotypes and synonymised *S. telescopica, T. longicauda, T. rufipes* with *S. ania*. Yang (1991) first reported the species in China mainland and re-described the female. Huang and Xiao (2005) re-described the female, stated the male colour and length and reported one male and one female from Liaoning Province, as well as one female from Beijing City; they illustrated the habitus, head, antenna and metasoma of the female individual. Gibson (2003) reported new distributional records in China, provided a key to the species of the genus and synonymised *T. silvae.* Sureshan (2005) reported new hosts and new distributional records in India, re-described the female and illustrated differences between *S. ania* and *S. feretrius*. Sureshan et al. (2013) reported new distributional records in India, with 10 males and 20 females parasitising *Clytocera chionospila*, with field photos of the female and larva.

#### Notes

The sexual dimorphism of this genus, which is representative and extreme in this family or even in Chalcidoidea, is seldom mentioned prior to this study: (1) the male is green (the female is blue); (2) the male body size is much smaller (the female can exceed 28 mm); (3) the male scape is half as long as head median length (female scape is as long as head median length (Fig. 2d); (4) 4^th^ -7^th^ tergites of male not elongate (4^th^ -7^th^ tergites of female distinctly elongate); (5) pronotal collar of male slightly longer than in female.

## Supplementary Material

XML Treatment for Solenura ania
